# Predictability of Delayed Visual Feedback Under Rubber Hand Illusion Modulates Localization but Not Ownership of the Hand

**DOI:** 10.3389/fpsyg.2021.771284

**Published:** 2021-11-12

**Authors:** Satoshi Shibuya, Satoshi Unenaka, Yukari Ohki

**Affiliations:** ^1^Department of Integrative Physiology, School of Medicine, Kyorin University, Tokyo, Japan; ^2^Department of Sport Education, School of Lifelong Sport, Hokusho University, Ebetsu, Japan

**Keywords:** body ownership, rubber hand illusion, multisensory integration, delayed visual feedback, predictability, body representation

## Abstract

The rubber hand illusion (RHI) is a perceptual illusion, whereby a fake hand is recognized as one’s own hand when a fake hand and felt real hand are stroked synchronously. RHI strength is mainly assessed using a questionnaire rating and proprioceptive drift (PD). PD is characterized by the proprioceptively sensed location of the participant’s own hand shifting toward the location of the fake hand after RHI. However, the relationship between the two measures of hand ownership and location remains controversial due to mixed findings: some studies report correlations between them, while others show that they are independent. Here, we demonstrated significant PD without RHI using delayed visual feedback. In this RHI study, video images of the fake hand were delivered to the subjects, and four delay intervals of visual feedback (80, 280, 480, and 680ms) were introduced. In four of six conditions, the delay interval was fixed throughout the condition. In the other two conditions, four delays were delivered in a predetermined order (i.e., serial condition; higher predictability) or in a pseudo-random order (i.e., random condition; low predictability). For the four conditions with a fixed delay, the questionnaire ratings and PD declined significantly when the delay interval exceeded *circa* 300ms. In both the serial and random conditions, no illusory ownership of the fake hand was reported in the questionnaire. In contrast, greater PD was found in the random condition but not in the serial condition. Our findings suggest that hand ownership and localization are caused by distinct multisensory integration processes.

## Introduction

A sense of body ownership is an experience of the body as a part of the self (“our body is our own”), which is of critical importance to self-consciousness (Gallagher, 2000). Many studies have examined body ownership using a RHI (Botvinick and Cohen, 1998): if a visually occluded subject’s own hand and a visible fake (rubber) hand are stroked in synchrony using paintbrushes, the subject perceives the fake hand as his/her own hand. In contrast, asynchronous visuo-tactile stroking weakens or abolishes the illusion. To estimate RHI strength, a questionnaire rating and proprioceptive drift (PD) have been frequently used as reliable measures in previous studies. PD is a behavioral phenomenon in which the proprioceptive position of the real hand shifts toward the fake hand after synchronous visuo-tactile stroking (synchronous condition) but not asynchronous stroking (asynchronous condition). Because the RHI strength from the questionnaire correlates with the PD magnitude, earlier studies assumed that hand ownership and proprioceptive localization of the hand have a common underlying process of multisensory integration (Botvinick and Cohen, 1998; Tsakiris and Haggard, 2005). However, several previous studies have indicated that the two components are composed of functionally different processes (Holle et al., 2011; Rohde et al., 2011; Cowie et al., 2013; Abdulkarim and Ehrsson, 2016; Radziun and Ehrsson, 2018; Filippetti et al., 2019; Matsumiya, 2019; Fiorio et al., 2020), although they have been reported to correlate. Rohde et al. (2011) found that greater PD without a sense of ownership over the fake hand occurred during intermittent asynchronous stroking but not during prolonged asynchronous stroking. Hence, they argued that PD was mainly caused by attenuation effects of prolonged asynchronous stimulation on visuoproprioceptive integration of the hand (e.g., Holmes et al., 2006), rather than by the facilitatory effects of synchronous stimulation. The longer asynchronous stroking is strong evidence against “unity assumption” (i.e., assumption that visual and proprioceptive sensations originate from the identical hand; Welch and Warren, 1980). In contrast, intermittent asynchronous stroking interrupts the accumulation of evidence, which leads to greater PD.

Recent theories of predictive coding postulate that human perception is strongly linked to continuous prediction of upcoming sensory signals (Clark, 2013). The view of the unity assumption fits into that of predictive coding, considering that unity assumption modulates the upcoming multisensory signals to be either integrated or not (Chen and Spence, 2017). Therefore, predictability of visual stimulation following tactile stimulation in asynchronous stroking may influence body perception and representation. The current study examined whether the predictability of visual feedback delay under RHI influences hand ownership (i.e., questionnaire) and localization (i.e., PD). In previous studies using RHI and delayed visual feedback, the delay interval was fixed throughout the condition, and results from several conditions with different delays were compared to explore how temporal discrepancy of visuo-tactile stimulation modulates RHI (Shimada et al., 2009, 2014). According to the previous procedure, our study established four conditions, where each delay (i.e., 80, 280, 480, or 680ms) was fixed. Moreover, there were two novel conditions in which the four delays were delivered in a predetermined order (i.e., serial condition) or in a random order (i.e., random condition). Consequently, the upcoming visual stimulation after tactile stimulation was highly predictable in the serial condition but not at all in the random condition. Similar to the view of Rohde et al. (2011), we hypothesized that low (unstable) predictability of delayed visual feedback under the random condition would disturb the accumulation of evidence against unity assumption, resulting in greater PD. In contrast, higher (more stable) predictability under the serial condition would lead to much smaller PD by attenuation effects of asynchronous stroking as usual. However, no illusory ownership of the fake hand would be found in either condition.

## Materials and Methods

### Subjects

In total, 28 healthy subjects (11 men and 17 women; mean age±standard deviation, 27.2±5.3years) participated. The subjects were blinded to the purpose of the experiment. All subjects were right-handed according to the Edinburgh Handedness Inventory (Oldfield, 1971). This study was approved by the Institutional Review Board at the Kyorin University School of Medicine and conducted according to the principles and guidelines of the Declaration of Helsinki. All subjects provided written informed consent prior to study participation in accordance with the institutional guidelines.

### Apparatus

**Figure 1:**
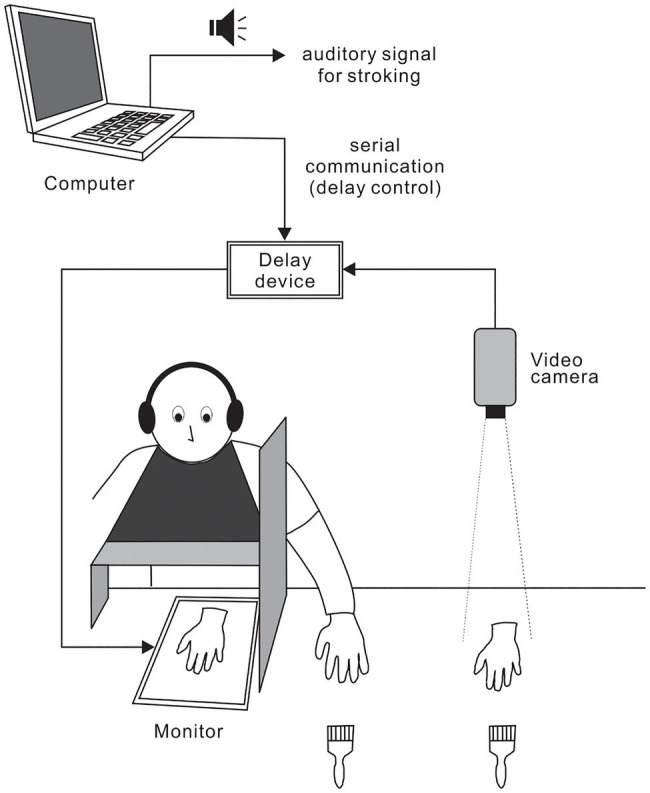
Experimental setup. Subject’s left hand and fake left hand were stroked simultaneously with paintbrushes to induce the rubber hand illusion (RHI). Video images from the top view of the fake hand were displayed on a monitor. Using a video delay device, four delay intervals (i.e., 80, 280, 480, or 680ms) were introduced. Delay intervals could be changed instantaneously by signals from a computer.

Subjects were comfortably seated in a chair and fitted with a latex glove on their left hand, which was placed at a predetermined position on a table. A 15-inch tablet monitor (On-lap 1503H, Gechic Corp., Taichung, Taiwan) was placed face-up on the table in front of the subjects (Figure 1). A wooden shelf was positioned approximately 16cm above the forearms of the subjects. Subjects were fitted with a black cape to conceal the space between the shelf and the subjects’ torsos. To prevent subjects from watching their own hand, a partition was placed between the monitor and each subject’s left hand. A life-sized artificial hand (i.e., fake hand) fitted with a latex glove was positioned next to the subject’s left hand. We recorded the top-view image of the fake hand using a full-HD video camera (60fps) and displayed the images on the monitor using a video delay device (VM-800HD, Sugioka system Corp., Osaka, Japan). Because the delay device was connected to a laptop computer (LB-J520S2, Mouse computer Co., Tokyo, Japan) *via* a serial port, the delay intervals of visual feedback could be changed instantaneously through signals from the computer. The distance between the fake hand image and the subject’s hand remained at 20cm throughout the experiment. To induce RHI, an experimenter stroked the fake hand and subject’s hand (digits 2, 3, and 4) simultaneously with two paintbrushes at *circa* 0.4Hz using auditory cues from the computer. The timing of auditory cues and serial port outputs were accurately controlled by Presentation 20.0 (Neurobehavioral Systems Inc., Berkeley, CA, United States). Four types of delays, including no artificial delay, were used. The inherent delay of our video system was *circa* 80ms. In addition, delays of 200, 400, and 600ms using the delay device were introduced to produce actual delay intervals of 280, 480, and 680ms, respectively. White noise (80dB) was played through headphones placed over the subjects’ plugged ears to mask brushing sounds.

### Procedure

Each subject performed six conditions (sessions) in a pseudo-random order. Each session comprised 64 visuo-tactile strokes (*circa* 160s). In four of the six conditions, each delay interval was fixed throughout the session (i.e., 80, 280, 480, and 680ms conditions). In addition, we established two novel conditions (i.e., the serial and random conditions), wherein each delay interval (80, 280, 480, and 680ms) was delivered using 16 strokes (64 strokes in total). In the serial condition, a sequence of 80-280-480-680-680-480-280-80ms was repeated eight times, whereas, the delay interval was pseudo-randomly assigned from 80, 280, 480, and 680ms in the random condition. All subjects were unable to notice switches in the delay interval because switching was implemented immediately before stroking.

Before and after each session (i.e., pre-test and post-test), the subjects were asked to judge the proprioceptive position of their own left hand in the following way. First, the subjects closed their eyes, and then the experimenter removed the partition and placed a black board with a ruler approximately 6cm over the subject’s hand. Then, the subjects opened their eyes and vocally reported the location of the middle fingertip of their unseen left hand using a ruler. The ruler was offset in each trial to prevent the influence of the response values from prior trials. Proprioceptive drift (PD) was calculated by subtracting the pre-test value from the post-test value in each session. After the post-test of proprioceptive judgment, subjects reported their subjective feelings during RHI induction in a questionnaire, which was employed in the original study by Botvinick and Cohen (1998). The questionnaire comprised seven items (Q1-7; Table 1). Subjects responded to all items using a 7-point Likert scale ranging from +3 (strongly agree) to −3 (strongly disagree). Seven items were classified into two categories: *ownership* (Q1-3) and *ownership control* (Q4-7). Subjects were allowed a 5-min rest between the sessions. All statistical analyses were performed using STATA, version 16.0 (Stat Corp., College Station, TX, United States).

**Table 1:** Questionnaire comprising seven items that were classified into two categories.

Category	Question: 7-point scale from −3 (disagree strongly) to +3 (agree strongly)
Ownership	1. It seemed as if I were feeling the touch of the paintbrush in the location where I saw the rubber hand touched.
	2. It seemed as though the touch I felt was caused by the paintbrush touching the rubber hand.
	3. I felt as if the rubber hand were my hand.
Ownership control	4. It seemed as if I might have more than one left hand or arm.
	5. It seemed as if the touch I was feeling came from somewhere between my own hand and the rubber hand.
	6. It felt as if my (real) hand were turning rubbery.
	7. It appeared (visually) as if the rubber hand were drifting towards my hand.

## Results

### Questionnaire Rating

**Figure 2:**
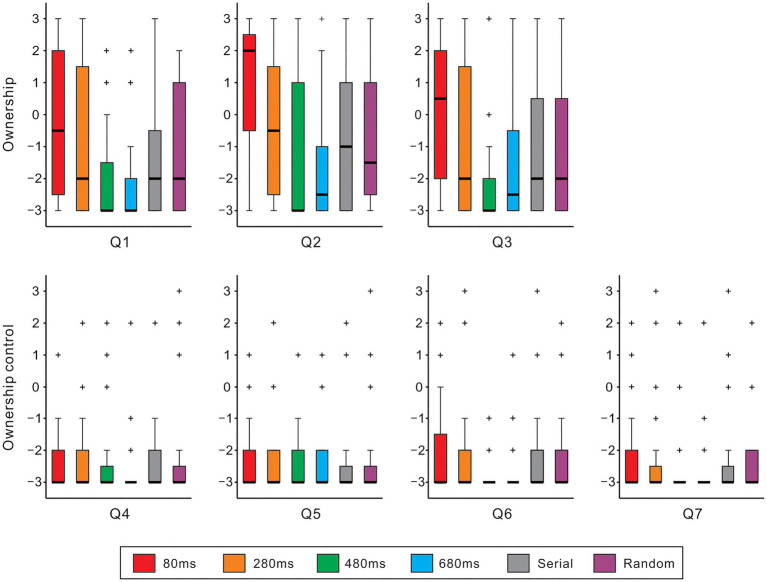
Questionnaire rating. Boxplots of questionnaire ratings of six experimental conditions with different colors are shown for *ownership* (top panels: Q1–Q3) and *ownership control* (bottom panels: Q4–Q7). Boxes and thick lines denote the interquartile ranges (IQRs) and medians, respectively. Whiskers show either additional data points or extend to 1.5×IQR. Small plus signs indicate outliers.

Figure 2 shows boxplots of the questionnaire ratings against different conditions for each item. The Friedman test demonstrated that the null hypothesis of equal medians across six conditions was rejected in all *ownership* items (Q1-3: *χ*^2^[5]>32.9, *ps*<0.01, all) and an *ownership control* item (Q6: *χ*^2^[5]=12.4, *p*<0.05). Across *ownership* items, the ratings of the 80ms condition were significantly greater than those of the 480ms, 680ms, serial, and random conditions (Q1: *ps*<0.05; Q2: *ps*<0.01; Q3: *ps*<0.01; Scheffe’s test). Under the 80ms condition, approximately half of the subjects (Q1: 46% [13/28]; Q2: 60% [17/28]; Q3: 50% [14/28]) showed positive responses (>0: affirmation). A one-sample *t*-test indicated that the rating was greater than zero in Q2 (mean=0.9; t[27]=2.2, *p*<0.05) but not in Q1 (−0.04) or Q3 (0.3; *ps*>0.5, both). Additionally, the mean rating across Q1-Q3 was not statistically larger than zero (mean=0.4; *p*>0.3). The rating of the 280ms condition was higher than that of the 480ms and 680ms conditions in Q1 (*ps*<0.05) and Q2 (*ps*<0.05). While the medians had the lowest score (−3) across all *ownership control* items, a significant difference between the 80ms and 480ms conditions was identified in the Q6 (*p*<0.05; Scheffe’s test).

### Proprioceptive Drift

**Figure 3:**
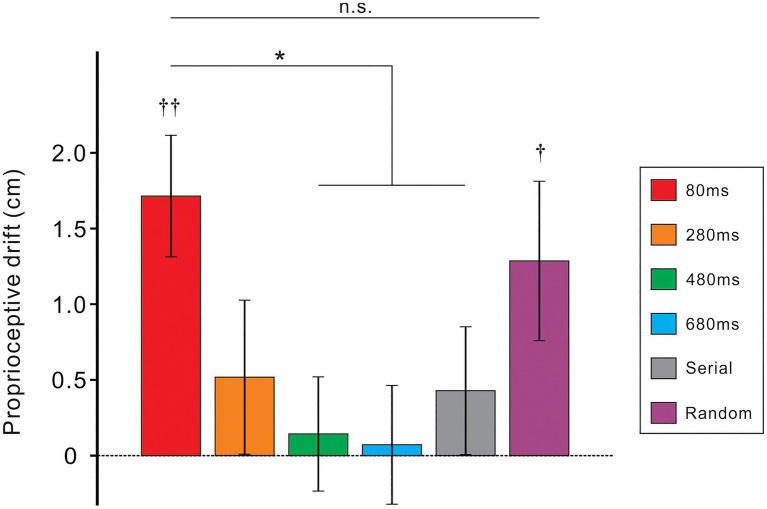
Mean proprioceptive drift (PD) for six experimental conditions with different colors. Vertical lines denote ±1.0 standard error of the mean (SEM). Significance differences from the value of the 80ms condition (Dunnett test): ^*^ps<0.05. Significance differences from zero (one-sample *t*-test): ^††^*p*<0.01, ^†^*p*<0.05.

Figure 3 shows mean PD against the six conditions. A one-way analysis of variance indicated a significant main effect [F(5, 135)=2.7, *p*<0.05; *η*^2^=0.09]. To compare between 80ms condition (nearly synchronous condition) and other five conditions, we performed a *post-hoc* Dunnett test. As a result, the PD of the 480ms, 680ms, and serial conditions significantly decreased compared to the 80ms condition (*ps*<0.05, all). Although the PD of the 280ms condition was quite small, the difference between the 80ms and the 280ms conditions did not achieve significant level (*p*=0.07). Whereas, there was no difference between the 80ms and random condition (*p*>0.5). The difference between the random and serial conditions was not statistically significant (*p*=0.14; one-sample *t*-test). We further tested whether the PD of each condition was statistically bigger than zero using a one-sample *t*-test. Results showed that the PD was significantly greater than zero in the 80ms (t[27]=4.3, *p*<0.01) and random conditions (t[27]=2.4, *p*<0.05) but not in the remaining conditions (*ps*>0.3, all).

Finally, we performed correlation analyses between the PD and ratings of *ownership* items (Q1-Q3) in the 80ms condition. Consequently, moderate positive correlations were found in all combinations (Q1: *r*=0.47; Q2: *r*=0.41; Q3: *r*=0.40; *ps*<0.05, all).

## Discussion

The current study examined how the predictability of visual feedback delay (i.e., temporal discrepancy between visual and tactile stimulation) under RHI affects ownership and perceived location of the hand. In the four conditions with fixed delays, *ownership* ratings showed that RHI was induced when nearly synchronous visuo-tactile stimulation was delivered in the 80ms condition, and longer delay intervals beyond 300ms abolished RHI (i.e., 480 and 680ms conditions). Similarly, the proprioceptive drift (PD) was attenuated as a function of delay intervals. In both the serial and random conditions, the subjects mostly denied RHI in the questionnaire. In contrast, PD was modulated by the predictability of delay intervals. Under the serial condition (higher predictability), the PD was significantly less compared to the 80m condition. However, greater PD was found under the random condition (low predictability), suggesting that PD occurred without illusory ownership of the fake hand. Our results support the view that ownership and localization of the hand are distinct components that result from separate multisensory integration processes. To the best of our knowledge, the current study is the first to uncover the distinct modulation of predictability of delayed visual feedback under RHI on ownership and localization of the hand.

Under the four conditions with fixed delays, the *ownership* ratings were clearly attenuated as a function of visual feedback delay. In the 80ms condition (i.e., nearly synchronous condition), half of the participants expressed positive responses for *ownership* ratings (52%: Q1-3), although the ratio was somewhat smaller than the standard RHI studies using a direct view of the fake hand (*circa* 70%; Lloyd, 2007; Kalckert and Ehrsson, 2014). When the delay intervals exceeded 300ms (i.e., 480ms and 680ms conditions), the subjects denied illusory ownership of the fake hand (*ownership* ratings < −2). The *ownership* ratings of the 280ms condition indicated intermediate values between 80ms and 480ms conditions. Questionnaire results suggest that synchronous visuo-tactile stimulation induces RHI, and they are basically consistent with the previous findings that RHI declines if a visuo-tactile temporal discrepancy of greater than 200–300ms is delivered (Shimada et al., 2009, 2014; Shibuya et al., 2019). In terms of PD, a one-sample *t*-test indicated that significant drifts occurred only in the 80ms condition. Moreover, the PD of the 480 and 680ms conditions was significantly less than that of the 80ms condition. These results are also in line with previous reports showing that PD decreases gradually (or linearly) by increasing visual feedback delay (Shimada et al., 2014; Shibuya et al., 2019).

A more important discovery is that the predictability of delayed visual feedback modulated the ownership and perceived localization of the hand differently. *Ownership* ratings were considerably low in both serial and random conditions (medians < −1, all). This result is unsurprising because the subjects experienced visual feedback with longer delays (i.e., 480 and 680ms; 50%) more frequently than the shortest delay (i.e., 80ms; 25%). Considering previous reports which state that just vision of the fake hand does not induce an illusory feeling of ownership (Holmes et al., 2006; Longo et al., 2008; Rohde et al., 2011), our findings suggest that synchronous visuo-tactile stimulation for a certain time ranging from tens of seconds (Ehrsson et al., 2004) to a few minutes (Shimada et al., 2014) is essential to induce an illusory ownership of the fake hand. Whereas, an obvious difference in PD was obtained between the two: the PD magnitude of the serial condition (higher predictability) was not statistically significant (*p*>0.3; one-sample *t*-test), and it was much less than the 80ms condition, while the subjects under the random condition produced greater PD, which was as large as that of the 80ms condition. The PD difference seems to be related to the effects of visuo-tactile stimulation on visuoproprioceptive (VP) integration of the hand. It is generally accepted that when the locations of the seen (vision) and felt hands (proprioception) are dissociated using a mirror or prism spectacle, our brain recalibrates proprioceptive representation of the hand according to visual dominance, regardless of ownership of the visual hand (VP integration; e.g., Holmes et al., 2006). A model of hand ownership proposed by Makin et al. (2008) assumed that synchronous visuo-tactile stimulation under RHI provides positive feedback on the processes of VP integration. According to this view, one explanation for our results is that updating of proprioceptive representation of the self-hand based on visual input was facilitated only when synchronous visuo-tactile stimulation (i.e., 80ms delay) was presented under the random condition. However, this possibility, which places emphasis on visuo-tactile synchrony, is probably unlikely because nearly synchronous stroking (80ms delay) was less frequent than asynchronous stroking (480ms and 680ms delays). The other explanation is that the PD difference was mainly caused by the function of visuo-tactile asynchronous stroking, rather than synchronous stroking. Rohde et al. (2011) found that intermittent asynchronous (spatially and temporally unrelated) stroking (10s×12) due to frequent measurements produced large PD as synchronous stroking, while infrequent measurements (120s×1) during prolonged asynchronous stroking produced smaller PD relative to synchronous stroking, as previously reported. Moreover, the PD of the vision-only condition (i.e., subjects watched the rubber hand without visuo-tactile stimulation) was as large as that of the synchronous stroking. This result also refutes the facilitatory effects of synchronous visuo-tactile stroking on PD. Based on these results, Rohde et al. (2011) modified the model of Makin et al. (2008) by arguing that asynchronous visuo-tactile stroking provides negative feedback on VP integration, resulting in a smaller PD. Specifically, they insisted that prolonged asynchronous stroking would serve as the strong evidence against unity assumption (Welch and Warren, 1980), which postulates that visual and proprioceptive sensations originate from the same hand. If two different senses are not perceived as belonging together, no VP integration (i.e., PD) will occur. Based on this view, intermittent asynchronous stroking disturbs the accumulation of evidence against unity assumption. Whereas, visuo-tactile integration is necessary for feelings of ownership. Our findings might be congruent with the findings of Rohde et al. (2011). That is, we presume that the low (unstable) predictability of delayed visual feedback under the random condition interferes with the accumulation of evidence against unity assumption, such as intermittent asynchronous stroking (Rohde et al., 2011), resulting in greater PD. In contrast, asynchronous visuo-tactile stimulation under serial conditions (higher and more stable predictability) would provide the stronger evidence against unity assumption. If this is the case, our findings might support a view that unity assumption (i.e., top-down modulation of multisensory perception) can be considered as a sort of prediction (Chen and Spence, 2017).

The fact that greater PD occurred without ownership of the fake hand in the random condition suggests a distinction between ownership and location of a body part, although moderate correlations between *ownership* ratings and PD were observed in the 80ms condition. Indeed, this view is supported by recent behavioral studies (Holle et al., 2011; Rohde et al., 2011; Cowie et al., 2013; Abdulkarim and Ehrsson, 2016; Radziun and Ehrsson, 2018; Filippetti et al., 2019; Matsumiya, 2019; Fiorio et al., 2020). For instance, Abdulkarim and Ehrsson (2016) showed that illusory ownership of the fake hand was not influenced, even when a participant’s hand position was mechanically moved toward or away from the fake hand during RHI induction. The perceived hand position could be strongly affected by the external manipulation of the hand position. These findings suggest that there was no causal link between the two components. From the perspective of computational models of multisensory integration, Matsumiya (2019) discovered that the model of optimal cue combination could explain the PD results well but not the results of hand ownership. Consequently, previous studies and our study support the possibility that ownership and localization of the hand arise from distinct multisensory integration processes in the brain. Neurophysiological findings have shown that different brain regions are associated with two components. An RHI study using functional magnetic resonance imaging (fMRI) demonstrated that the activities of the premotor cortex (PM) and the inferior parietal lobe (IPL) were modified between synchronous and asynchronous conditions (Ehrsson et al., 2004). They concluded that neural activity in the PM reflected the feeling of hand ownership, while “it is still somewhat unclear whether the activity in the intraparietal cortex reflects the feeling of ownership *per se*, because we only detected a trend for illusion-related activity in this region.” A related fMRI study also found that hand-centered remapping to the fake hand in the PM was closely related to hand ownership, while remapping in the IPL (especially, supramarginal gyrus) reflected the PD changes (Brozzoli et al., 2012). In fact, repetitive transcranial magnetic stimulation over the IPL attenuates PD but not hand ownership (Kammers et al., 2009). Therefore, different characteristics of multisensory integration in the PM and IPL might contribute to the current findings that predictability of delayed visual feedback influences ownership and localization of the hand differently.

Our study has some limitations. First, differences in delay intervals between a stroke and subsequent stroke did not remain constant between the serial and random conditions. The difference was either 0 or 200ms in the serial condition (i.e., 80-280-480-680-680-480ms…), whereas it ranged from 0 to 600ms (e.g., 0, 200, 400, or 600ms) in the random condition. Thus, it is possible that the variability of the delay differences affected the PD results. Second, while our findings support the view that asynchronous visuo-tactile stroking inhibits the occurrence of PD, we cannot refute the possibility that synchronous stroking enhanced the PD in the 80ms condition, because we did not examine the PD magnitude only due to VP integration (i.e., vision-only condition). Third, the inherent delay of the video system was 80ms. A prior RHI study reported that most subjects (>90%) did not detect temporal inconsistency between visual and tactile stimulation by 100ms (Shimada et al., 2014). Nevertheless, a slight delay of 80ms might affect the questionnaire and PD results. Fourth, the experimenter stroked the subject’s and fake hands simultaneously across all conditions. It is natural that there was some variability in timing of manual stroking (i.e., slight discrepancy of visuo-tactile stimulation). Nevertheless, we believe that such a variability had little effect on current results, because timing variability of synchronous stroking would much less than variability of visual feedback delay as mentioned above. Finally, it is unclear whether our findings can be replicated using different experimental procedures. For instance, we used a specific procedure to assess PD (i.e., visual judgment with a ruler). However, our group also found that PD magnitude was dependent on measuring methods (e.g., manual reaching or contralateral matching; Shibuya et al., 2017). The RHI can be induced not only by visuo-tactile synchrony but also by visuo-motor synchrony (i.e., moving RHI; Kalckert and Ehrsson, 2014). Thus, the effects of predictability of visual feedback delays might be modulated in different ways to induce the RHI. Although these issues need to be addressed in future studies, our findings provide useful information about asynchronous visuo-tactile stroking, which is used as a control condition in RHI studies.

## Data Availability Statement

The raw data supporting the conclusions of this article will be made available by the authors, without undue reservation.

## Ethics Statement

The studies involving human participants were reviewed and approved by the Institutional Review Board of Kyorin University School of Medicine. The patients/participants provided their written informed consent to participate in this study.

## Author Contributions

All authors conceived and designed the experiment and discussed the results. SS performed the experiment, analyzed the data, and wrote the first draft. All authors approved the final version of the manuscript.

## Funding

This work was supported by the Japan Society for the Promotion of Science, KAKENHI (Grant Nos. 20K11423 to SS and 20K07714 to YO).

## Conflict of Interest

The authors declare that the research was conducted in the absence of any commercial or financial relationships that could be construed as a potential conflict of interest.

## Publisher’s Note

All claims expressed in this article are solely those of the authors and do not necessarily represent those of their affiliated organizations, or those of the publisher, the editors and the reviewers. Any product that may be evaluated in this article, or claim that may be made by its manufacturer, is not guaranteed or endorsed by the publisher.
